# Effectiveness of Oncological Physiotherapy on Shoulder Dysfunction After Cervical Lymph Node Dissection in Head and Neck Cancer: A Pilot Randomized Controlled Trial

**DOI:** 10.3390/medicina61091636

**Published:** 2025-09-10

**Authors:** Raquel Pérez-García, Vanesa Abuín-Porras, Daniel Pecos-Martín, Carlos Romero-Morales

**Affiliations:** 1 University of Alcalá, Department of Nursing and Physiotherapy, Faculty of Medicine and Health Sciences, 28801 Alcalá de Henares, Madrid, Spain; raquel.perez@universidadeuropea.es (R.P.-G.); daniel.pecos@uah.es (D.P.-M.); 2 Universidad Europea de Madrid, Faculty of Medicine, Health and Sports, Department of Physiotherapy, 28670 Villaviciosa de Odón, Madrid, Spain; carlos.romero@universidadeuropea.es; 3 Grupo de Investigación Fisioterapia y Dolor, Departamento de Enfermería y Fisioterapia, Universidad de Alcalá, 28801 Alcalá de Henares, Madrid, Spain

**Keywords:** head and neck cancer, oncological physiotherapy, shoulder pain, shoulder dysfunction

## Abstract

*Background and Objectives*: Shoulder dysfunction is a frequent complication after cervical lymph node dissection in patients with head and neck cancer (HNC), leading to pain, reduced mobility, and impaired quality of life. Physiotherapy programs that include strength exercises have shown benefits in managing these sequelae, but the potential added value of neurodynamic mobilization techniques (NDMTs) remains unclear. This pilot randomized controlled trial was designed to examine whether a NDMTs program improves pain and shoulder-related function in HNC survivors with shoulder dysfunction, assessing trajectories during treatment and at short-term follow-up. *Materials and Methods*: A pilot, assessor-blinded, randomized, parallel-group clinical trial was conducted with 20 participants who had undergone HNC surgery and exhibited shoulder dysfunction. Participants were randomized to either a control group (strength exercises alone) or an experimental group (strength exercises plus NDMTs). Outcomes were assessed at baseline, mid-term (1 week), post-treatment, and 3 months post-treatment. The primary outcome was quality of life measured by the QLQ-H&N35 questionnaire. Secondary outcomes included pain intensity (VAS), disability (DASH), and handgrip strength. *Results:* Significant improvements were observed in the experimental group for all primary and secondary outcomes. The experimental group demonstrated improved quality of life (*p* = 0.009), lower pain intensity (*p* < 0.001), reduced disability (*p* < 0.001), and increased handgrip strength. Interaction effects for time and group were significant across multiple measures, favoring the NDMTs group. *Conclusions*: NDMTs are a promising addition to strength programs for improving shoulder dysfunction outcomes in HNC patients, with implications for both clinical practice and future research. Registered in ClinicalTrials: NCT05604235 prior to recruitment.

## 1. Introduction

Head and neck cancer (HNC) encompasses malignancies of the oral cavity, pharynx, and larynx that frequently require multimodal treatment [1,2,3,4]. Cervical lymph node dissection (CLND) is common in the oncologic management of HNC and places the spinal accessory nerve (SAN) at risk of traction, compression, or partial transection. Even when the SAN is anatomically preserved, neuropraxia and postoperative scarring can impair its function. The resulting trapezius and sternocleidomastoid weakness, altered scapulothoracic mechanics, and pain contribute to a characteristic shoulder dysfunction after CLND that includes limited range of motion—particularly abduction—scapular winging, and activity limitations that diminish quality of life [5,6,7,8].

Rehabilitation is central to addressing these sequelae. Strengthening of the scapular stabilizers and shoulder girdle, mobility and postural retraining, and progressive functional exercise are recommended and have shown benefits for pain and function in this population, although effect sizes and protocols vary across studies [9,10]. However, surgery and adjuvant radiotherapy can also induce neural mechanosensitivity and fibrosis along the neural interfaces of the accessory nerve and brachial plexus. In this context, techniques that specifically target the mobility and load tolerance of neural tissues may provide additional benefit beyond conventional strengthening alone.

Neurodynamic mobilization techniques (NDMTs) are manual therapy procedures designed to restore the excursion and mechanical interface of peripheral nerves through graded positioning, tensioning, and sliding [11]. In musculoskeletal conditions, NDMTs have been associated with short-term reductions in pain and improvements in range of motion and function. Recently, Joshi et al. reported that women with breast cancer treated with neural tissue mobilization showed significant decreases in pain and improved shoulder range of motion [12].

A search of the literature reveals no clinical trials evaluating neurodynamic mobilization techniques (NDMTs) specifically for shoulder dysfunction following cervical lymph node dissection in head and neck cancer (HNC) patients. A recent systematic review identified progressive resistance training as effective but did not include NDMTs among the studied interventions [9]. Similarly, Cochrane findings reported only on conventional exercise therapies, with no trials involving neural mobilization [13]. In contrast, NDMTs have demonstrated benefit in comparable oncologic contexts, such as breast cancer, where they led to improvements in shoulder mobility and pain reduction after axillary dissection [12]. These findings support the rationale for investigating NDMTs in the HNC population.

Therefore, the objective of this pilot randomized controlled trial was to determine whether adding NDMTs to a conventional program improves shoulder dysfunction after CLND in patients with HNC. We hypothesized that, compared with strengthening alone, the combined program would yield greater improvements in patient-reported outcomes and shoulder function across the treatment period and short-term follow-up.

## 2. Materials and Methods

**Figure 1:**
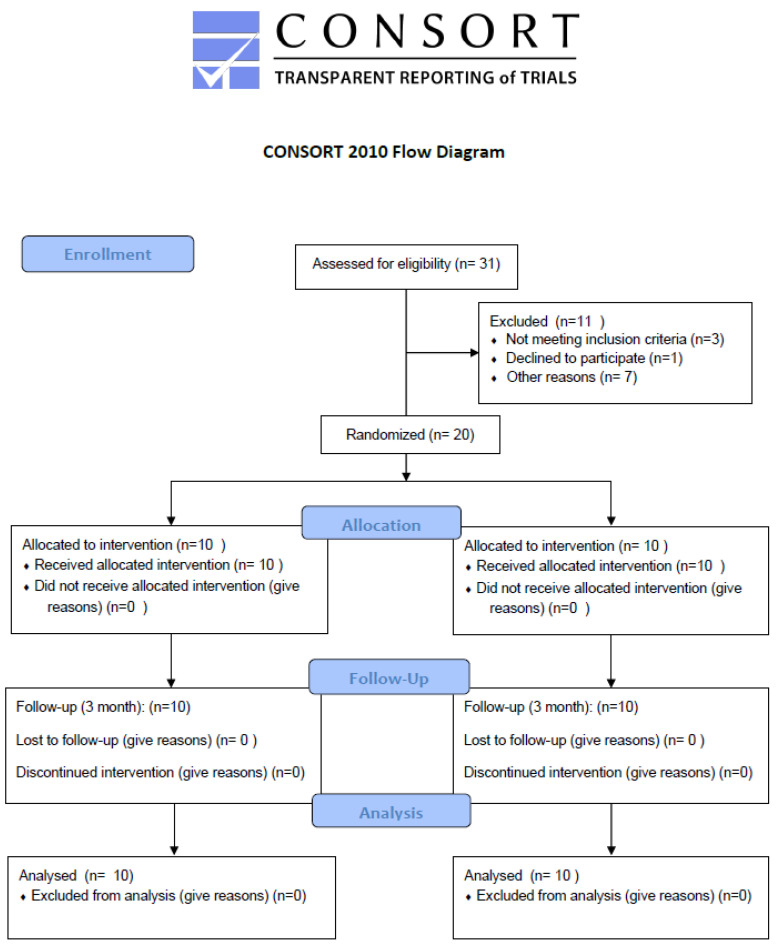
CONSORT 2010 Flow Diagram.

This study was conducted as a pilot randomized controlled trial, with the primary aim of evaluating feasibility and preliminary clinical effects of a physiotherapy program incorporating neurodynamic mobilization techniques. It followed the CONSORT (Consolidated Standards of Reporting Trials) extension for clinical trials [14] in accordance with the Declaration of Helsinki [15]. This study compared, with a 3-month follow-up, the effect of the inclusion of a neurodynamic mobilization intervention compared with a strength program on functional and psychosocial outcomes in patients surgically treated for head and neck cancer with shoulder dysfunction. The study design was approved on 25 February 2022 by Complejo Hospitalario Universitario de Canarias, Spain (CHUNSC_2021_91) and the trial was registered prior to recruitment on 3 November 2022 (ClinicalTrials: NCT05604235). The selection process is outlined in Figure 1.

### 2.1. Participants

Participants with shoulder dysfunction after HNC surgery, diagnosed by an oncologist were enrolled in the study. The study sample consisted of individuals between 18 and 80 years of age, with a diagnosis of head and neck cancer and treated with cervical lymph node resection between levels II and V, having shoulder pain and dysfunction. Subjects were excluded if they had bone metastases, lymph node dissection at cervical level I, or they had previous shoulder injuries.

We conducted an assessor-blinded randomized trial. Because the nature of the intervention prevented blinding of participants and treating therapists, only outcome assessors were masked to group assignment. Allocation was concealed with sequentially numbered, opaque, sealed envelopes prepared and administered by an independent researcher who had no role in screening, treatment delivery, or outcome assessment.

### 2.2. Outcomes

All outcome measures were administered by a blinded assessor at baseline assessment (pretreatment), mid-term (1 week), at treatment completion (3 months), and 3 months post-treatment completion (long term follow-up).

The data regarding the gender, age, level of cervical lymph node dissection, upper limb affected of the participants were recorded during the face-to-face interviews on a pre-prepared evaluation form.

Primary outcome (quality of life). Quality of life was measured with the QLQ-H&N35 questionnaire [16]. The QLQ-H&N35 is intended to be administered alongside the core QLQ-C30 [17] and has been validated in head and neck cancer populations, including a Spanish version with demonstrated validity and reliability [17]. The module refers to the period “during the past week,” and its format parallels that of the core questionnaire. Items 1–30 use four-point Likert-type responses (“not at all,” “a little,” “quite a bit,” “very much”), while Items 31–35 employ a “no/yes” format. Scores are linearly transformed to 0–100, where higher values indicate greater symptom burden or problems, consistent with the scoring of the QLQ-C30 symptom scales and single items. The Spanish version of the QLQ-H&N35 has also shown evidence of validity and reliability [18].

Pain intensity. Pain intensity was assessed using a visual analog scale (VAS) [19]. The VAS consisted of a 100-mm horizontal line anchored by the descriptors “no pain” and “worst pain ever.” Participants were instructed to draw a vertical mark at the point that best represented their current pain intensity. The VAS is a valid and reliable unidimensional measure for both acute and chronic pain and is suitable for use in adults diagnosed with head and neck cancer [20].

Upper-limb disability. Disability related to shoulder problems was evaluated with the Disabilities of the Arm, Shoulder and Hand (DASH) questionnaire [21]. The DASH is a 30-item self-report instrument that assesses the ability to perform specific upper-extremity activities. Patients rate difficulty and interference with daily life on a 5-point Likert scale, and the instrument has demonstrated validity (Pearson r > 0.70) and reliability (ICC = 0.96) [21]. Higher scores denote greater disability and severity, whereas lower scores indicate less disability.

Neurodynamic test. The neurodynamic test was administered with the patient seated [22]. Participants performed active cervical flexion, rotation, and side-bending toward the unaffected side while the examiner applied shoulder depression on the affected side. The test was classified as positive when pain, paresthesia, or abnormal tension was reported [22].

Skeletal muscle function. Skeletal muscle performance was examined using a handgrip strength test [23]. Handgrip strength was measured with a JAMAR^®^ handgrip dynamometer. The patient positioned the elbow at 90° of flexion with the wrist in neutral and then produced a maximal grip effort. Three trials were recorded, and the average of the three measurements was used as the handgrip strength value [23].

### 2.3. Interventions

Participants in both groups underwent 16 therapy sessions over eight weeks (two sessions per week). Control group (strengthening): Ten strengthening exercises primarily targeting the trapezius and sternocleidomastoid were performed with 2-kg weights, 20 repetitions per exercise, each session. The exercises included shoulder flexion and abduction with the elbow extended; scapular retraction/protraction in standing; bent-over row sequence without abduction; prone horizontal abduction with scapular retraction; seated scapular retraction with shoulders at 90° abduction and elbows at 90°; resisted ipsilateral lateral flexion and rotation of the cervical spine; an upright row with elastic band; and prone external rotation at 90° abduction. A complete step-by-step protocol (positions, cues, and execution) is provided in Supplementary Material S1.

Participants allocated to the neurodynamic group received neurodynamic mobilizations for accessory spinal nerve in addition to conventional treatment, performed by the same physiotherapist (Supplementary Material S1).

The NDMTs were performed with the patient in lateral decubitus on the unaffected side, the cervical spine was placed in flexion and a retraction of the scapula was performed. From this position, the patient was asked to slowly move his neck increasing flexion and up to neutral position, at the same time the physiotherapist mobilized the arm towards slight shoulder distraction and scapula depression [22]. The technique was performed for 10 min (Supplementary Material S1).

### 2.4. Statistical Analysis

Statistical analyses were conducted using RStudio (v1.4; RStudio, PBC, Boston, MA, USA) and IBM SPSS Statistics (v25; IBM, Armonk, NY, USA). Baseline descriptive summaries and independent-samples *t*-tests were calculated. Assumptions of normality were assessed with the Shapiro–Wilk test and inspection of histograms; a *p*-value > 0.05 was taken to indicate a normal distribution, after which parametric tests were applied, and otherwise non-parametric tests were used. To verify the validity of the experimental design, baseline between-group comparisons were performed to confirm the absence of pre-existing differences (independent-samples *t*-test).

To analyze outcomes at baseline, mid-term, final, and 3-month assessments, we fit a mixed linear model for repeated measures, specifying subject-level random effects by identifying each participant. This specification allowed us to distinguish changes attributable to individual variability from those due to group differences. We reported the intraclass correlation coefficient (ICC) and the percentage of variance, together with R-squared and conditional R-squared values, to evaluate variation in individual responses. Fixed factors included time (baseline, mid-term, post) and intervention (groups) to model intra- and inter-subject effects. Bonferroni-adjusted post hoc pairwise comparisons were applied. Effect sizes were expressed as partial eta squared (ηp^2^), interpreted as small (0.01), medium (0.06), and large (0.14). Statistical significance was set at *p* < 0.05 with α = 0.05 (95% confidence interval) and a desired power of 80% (β = 0.20).

## 3. Results

Twenty participants were randomized (10 per group). Cervical dissection levels were II–V (50%), III–V (30%), and IV–V (20%). The affected side was left in 55%. A total of 65% received surgery plus radiotherapy, 30% surgery only, and 5% surgery plus chemotherapy. All participants were positive on the neurodynamic test at baseline. Table 1 shows the groups’ sociodemographic data values, with no statistically significant differences between them. Moreover, dependent variables did not report significant baseline differences.

Considering Table 2, the time factor reported significant differences for all the dependent variables. Time × group interaction shows significant differences (*p* < 0.05) for pain intensity (F = 25.11; P = 0.001), test neuro (F = 6.92; P = 0.001), HN35 (F = 4.23; P = 0.009), PainHN35 (F = 7.753; P = 0.001) and DASH (F = 36.2; P = 0.001) variables in favor of the experimental group.

In addition, mixed linear models reported that pain intensity [ICC = 0.610, (R^2^_m_ = 0.663, R^2^_c_ = 0.868)], strength [ICC = 0.813 (R^2^_m_ = 0.403, R^2^_c_ = 0.888)] and PainHN35 [ICC = 0.771 (R^2^_m_ = 0.262, R^2^_c_ = 0.831)] vary in their proportion of explained variance for the dependent variable, considering the random effects.

## 4. Discussion

The main aim of this study was to determine the effects of adding a neurodynamic mobilization intervention into a multimodal treatment approach on management of shoulder dysfunction in patients treated with head and neck cancer surgery.

In this pilot randomized trial, adding neurodynamic mobilization to a conventional program was associated with improvements in pain, disability, and head-and-neck–specific quality of life compared with strengthening alone. In this line, the significant improvements in pain intensity, shoulder functionality (DASH score), and quality of life (NH35) between the experimental and control groups suggest that NDMTs could be a valuable addition in oncological physiotherapy programs.

Our primary outcome showed a significant group×time interaction favoring NDMTs for the QLQ-H&N35 (*p* = 0.009), indicating a greater reduction in symptom burden vs. strengthening alone (higher H&N35 scores = worse symptoms). To aid interpretation, we considered QLQ-H&N35 minimal important differences (MIDs), where 10-point changes are typically clinically relevant [15]. Although this is a pilot study, the direction and model estimates suggest a clinically meaningful advantage for the experimental arm, consistent with the validated properties of the H&N35 [17,18].

The benefits observed with NDMTs may be explained by their capacity to reduce neural mechanosensitivity and restore normal nerve excursion, particularly in the spinal accessory nerve affected by cervical lymph node dissection. These techniques improve axoplasmic flow, reduce intraneural edema, and decrease adverse mechanical tension, which together may lower nociceptive input and enhance neuromuscular function [24,25,26,27,28]. In turn, this can normalize scapulothoracic rhythm and alleviate compensatory movement patterns that contribute to pain and disability [5,7]. These mechanisms align with findings in other cancer populations—such as breast cancer patients—where NDMTs led to improved shoulder mobility and reduced pain following axillary dissection [12,24]. In combination with conventional physiotherapy, this can improve daily activities and social participation captured by the H&N35 [27,28]. Moreover, in HNC survivors with spinal accessory nerve injury, better shoulder/neck function is associated with higher quality of life [29]. Together, these mechanisms outline a plausible pathway from NDMTs leading to less pain/dysfunction and, consequently, better QoL.

Regarding the findings in the reduction in pain intensity, the NDMTs group reported significantly lower pain levels in VAS scale. This aligns with prior studies in other cancer populations, such as breast cancer patients, where the application of NDMTs have been shown improvements in pain intensity [24]. Joshi et al., found in their study that neural mobilization was found to significantly reduce sensory-motor impairments and pain associated with cancer-related conditions such as lymphedema [12]. This decrease in pain intensity could be attributed to the improved nerve mobility and reduction in neural tension, key mechanisms underlying neurodynamic approaches, as reported in multiple studies of musculoskeletal dysfunction [25,26,27] González-Matilla et al., in their systematic review conclude that neurodynamic mobilization significantly improves pain and function in musculoskeletal conditions by enhancing nerve mobility and reducing neural tension [30].

Considering the improvements in shoulder function, the NDMTs group exhibited enhanced motor function recovery, which supports findings from other studies related to the benefits of resistance exercises and physiotherapy interventions for shoulder rehabilitation in cancer patients [29,30]. Several authors have shown that progressive resistance training significantly improve range of motion (ROM), and enhance functional outcomes in patients undergoing cancer-related surgeries. Although most scientific literature focuses in shoulder rehabilitation after axillar lymphadenectomy in breast cancer [31,32,33], there are also some studies conducted in shoulder dysfunction in HNC patients [9]. Our results support the addition of NDMTs to these strength protocols.

Regarding strength, there were no significant differences between the groups. Since both of them performed a strength exercise program it is likely that both experienced improvements in strength, leading to similar outcomes and reducing the likelihood of a statistically significant difference between them. In addition, the ICC value (0.813) suggests that strength changes depended more on individual differences (ICC = 0.813) rather than the treatment alone.

Shoulder dysfunction related with HNC surgery has a significant impact on daily life, including activities of daily living, social interactions, and quality of life [29]. Therefore, the present study demonstrated that the addition of NDMTs not only improves the physical outcomes but also enhance psychosocial well-being. This aligns with findings from prior studies, which suggest that improvements in physical function are associated with better psychosocial health and increased overall life satisfaction in cancer survivors [34,35]. Moreover, shoulder pain and dysfunction in the general population are linked to significantly reduced health-related quality of life and increased psychological distress [36,37]. The findings of the present study emphasize the necessity of comprehensive physical therapy and the importance of sustained exercise programs to maintain both physical and mental well-being post-surgery.

### 4.1. Limitations

This study has several limitations that should be acknowledged. First, the small sample size (n = 20) and pilot design limit the statistical power and generalizability of the findings, although they do provide a foundation for future research. Second, the short follow-up duration (3 months post-treatment) may not fully capture long-term outcomes or the sustainability of improvements in shoulder function and quality of life. Third, the intervention protocol was standardized across participants, which may not reflect individualized clinical practice or account for patient-specific variability in response to treatment. Additionally, participants and the treating physiotherapist could not be blinded, which may introduce performance or response bias; we mitigated detection bias through assessor blinding and matched session frequency/duration across groups Finally, the absence of objective measures of nerve function (e.g., electromyography or nerve conduction studies) limits mechanistic conclusions. Future larger-scale trials with extended follow-up and more robust neurophysiological assessments are warranted to validate and expand upon these preliminary findings.

### 4.2. Future Lines

Further work should delineate the optimal dosage, frequency, and duration of neurodynamic mobilization, and evaluate its comparative and additive benefits against established rehabilitation approaches. Larger, adequately powered multicenter trials with longer follow-up are needed to determine whether these effects translate into sustained improvements in pain, function, and quality of life among cancer survivors.

### 4.3. Practical Applications

The findings of the present study suggest that NDMTs could be effectively integrated into standard physiotherapy protocols for patients experiencing shoulder dysfunction after HNC surgery. An early implementation of these techniques in the postoperative period may prevent the progression of shoulder dysfunction, improving recovery and reducing long-term disability.

## 5. Conclusions

Neurodynamic mobilization techniques (NDMTs) significantly improve pain, shoulder function, and quality of life in patients with shoulder dysfunction after head and neck cancer surgery. Integrating NDMTs into oncological physiotherapy protocols may enhance recovery and prevent long-term disability. These results are preliminary and require confirmation in larger, multicenter studies with longer follow-up.

## Figures and Tables

**Table 1:** Sociodemographic features of the sample.

Data	Experimental (n = 10)	Controls (n = 10)	*p*-Value (*t*-Test)
Age, years mean ± SD median (minimum–maximum)	59.9 ± 11.4 57.5 (43/85)	55.6 ± 13.0 55 (35–72)	0.443
Weight, kilograms mean ± SD median (minimum–maximum)	72.4 ± 12.4 73.5(46–89)	74.3 ± 14.4 72(54–99)	0.756
Height, meters mean ± SD median (minimum–maximum)	1.69 ± 0.08 1.69 (1.52–1.80)	1.72 ± 0.09 1.71(1.56–1.88)	0.439
BMI, k/m^2^ mean ± SD median (minimum–maximum)	25.1 ± 3.3 24.9(19.9–30.1)	24.8 ± 3.2 24.5(19.5–30.8)	0.817
Sex, masculine/feminine	6/4	7/3	N/A

Abbreviations: BMI = Body Mass Index.

**Table 2:** Mixed linear model to assess the time and groups factors for pain intensity, test neuro, strength, HN35, painHN35 and DASH.

					Fixed Effects	
Outcome Measure	Time Point	Experimental n = 10	Control n = 10	ICC, (R^2^_m_, R^2^_c_)	Time F (Df); P (*η_p_*^2^)	Group × Time F (Df); P (*η_p_*^2^)
Pain intensity				ICC = 0.610, (R^2^_m_ = 0.663, R^2^_c_ = 0.868)	F (1, 3) = 89.41; P = 0.001 (0.832)	F (1, 3) = 25.11; P = 0.001 (0.582)
	Baseline	6.80 ±2.44	6.10 ± 1.66			
	Middle–Term	5.50 ± 1.9	5.70 ± 1.5			
	Final	1.00 ± 1.2	4.80 ± 1.5			
	Follow up	0.40 ± 0.5	3.70 ± 1.16			
Test Neuro				ICC = 0.103 (R^2^_m_ = 0.661, R^2^_c_ = 0.696)	F (1, 3) = 42.00; P = 0.001 (0.700)	F (1, 3) = 6.92; P = 0.001 (0.278)
	Baseline	1.00 ± 0.0	1.00 ± 0.0			
	Middle–Term	0.90 ± 0.0	1.00 ± 0.0			
	Final	0.10 ± 0.3	0.80 ± 0.4			
	Follow up	0.40 ± 0.0	0.40 ± 0.0			
Strength				ICC = 0.813 (R^2^_m_ = 0.403, R^2^_c_ = 0.888)	F (1, 3) = 76.36; P = 0.001 (0.809)	F (1, 3) = 0.393; P = 0.759 (0.021)
	Baseline	19.0 ± 11.6	26.7 ± 9.9			
	Middle–Term	26.3 ± 10.3	32.8 ± 9.0			
	Final	35.5 ± 10.8	43.5 ± 9.2			
	Follow up	37.3 ± 9.5	45.3 ± 6.9			
HN35				ICC = 0.010 (R^2^_m_ = 0.977, R^2^_c_ = 0.977)	F (1, 3) = 1127.2; P = 0.001 (0.984)	F (1, 3) = 4.23; P = 0.009 (0.190)
	Baseline	1.09 ± 0.2	1.33 ± 0.2			
	Middle–Term	36.7 ± 5.3	41.5 ± 4.8			
	Final	1.08 ± 0.1	1.22 ± 0.1			
	Follow up	0.03 ± 0.0	0.03 ± 0.0			
PainHN35				ICC = 0.771 (R^2^_m_ = 0.262, R^2^_c_ = 0.831)	F (1, 3) = 32.933; P = 0.001 (0.647)	F (1, 3) = 7.753; P = 0.001 (0.301)
	Baseline	29.31 ± 17.5	21.2 ± 17.7			
	Middle–Term	20.8 ± 15.4	17.5 ± 15.8			
	Final	5.78 ± 4.4	14.1 ± 14.0			
	Follow up	3.0 ± 2.0	10.8 ± 13.7			
DASH				ICC = 0.413, (R^2^_m_ = 0.925, R^2^_c_ = 0.956)	F (1, 3) = 505.4; P = 0.001 (0.966)	F (1, 3) = 36.2; P = 0.001 (0.668)
	Baseline	56.5 ± 10.9	46.6 ± 5.0			
	Middle–Term	36.7 ± 5.3	41.5 ± 4.8			
	Final	1.8 ± 2.6	17.3 ± 5.4			
	Follow up	0.7 ± 1.5	14.7 ± 4.3			

## Data Availability

Data are available as supplementary material.

## Supplementary Materials

The following supporting information can be downloaded at: https://www.mdpi.com/article/10.3390/medicina61091636/s1, Supplementary Material S1: TIDieR Checklist—Intervention Summary.

## Abbreviations

The following abbreviations are used in this manuscript:

HNC	Head and Neck Cancer
NDMTs	Neurodynamic Mobilization Techniques
QLQH&N35	Quality of life Head & Neck
VAS	Visual Analog Scale
DASH	Disabilities of the Arm, Shoulder and Hand
