# Supplementary material for: Effectiveness of Oncological Physiotherapy on Shoulder Dysfunction After Cervical Lymph Node Dissection in Head and Neck Cancer: A Pilot Randomized Controlled Trial
Source: Medicina (Kaunas). 2025 Sep 10;61(9):1636. doi: 10.3390/medicina61091636 (PMC12471930; doi:10.3390/medicina61091636)
Supplement: Supplementary file 1 [file medicina-61-01636-s001.zip › medicina-3807353-supplementary.pdf]

Supplementary Material S1

TIDieR Checklist – Intervention Summary

TIDieR item	Control arm	Experimental arm
**1) Brief name**	Progressive strengthening program for cervical–scapular muscles (focus on trapezius and sternocleidomastoid).	Conventional Physiotherapy +Accessory spinal nerve neurodynamics.
**2) Why (rationale/theory)**	Strengthen trapezius and sternocleidomastoid to address cervical–scapular function.	Improve joint/muscle balance of the scapulohumeral complex and address neural mechanosensitivity via accessory spinal nerve neurodynamics.
**3) What — materials**	2-kg dumbbells; medium-resistance elastic band.	Stretcher or height-matched support; mirror for visual feedback.
**4) What — procedures (content)**	10 strengthening exercises; 20 reps each:<br>1) Standing shoulder flexion with 2 kg, elbow extended.<br>2) Standing shoulder abduction with 2 kg, elbow extended.<br>3) Standing scapular retraction/protraction with 2 kg each hand; avoid shoulder elevation.<br>4) Standing, trunk ~45° flexed, contralateral hand supported; 2 kg row: scapular retraction, then elbow flexion (no abduction).<br>5) Standing, trunk flexed; pendulum → horizontal abduction + scapular retraction with 2 kg.<br>6) Sitting: shoulders abducted ~90° with slight flexion; elbows 90°; scapular retraction (no adduction or extra elbow flexion).<br>7) Sitting: ipsilateral side-bending of neck against hand resistance.<br>8) Sitting: cervical rotation against hand resistance.	A) Passive mobilizations by physiotherapist:<br>1) Shoulder extension with elbow flexion (side-lying on healthy side) with scapular depression.<br>2) Shoulder flexion with elbow extended; scapula fixed (side-lying).<br>3) Circumduction with distraction (side-lying), elbow extended; scapula fixed.<br>4) Adduction/abduction + external rotation with elbow flexed (side-lying); mobilize from elbow while scapula fixed.<br>5) Tissue stretching & gentle glenohumeral decoaptation with arm supported.<br><br>B) Active kinesitherapy (10–12 reps each; mirror to avoid compensations):<br>1) Standing shoulder flexion, elbow extended.<br>2) Standing shoulder abduction, elbow extended.

	9) Standing: upright row with medium elastic band from feet to chest/chin level, elbows slightly above shoulders.<br>10) Prone: shoulder abducted ~90°, hand pronated; external rotation with 2 kg.	3) Standing scapular retraction/protraction (no elevation).<br>4) Standing, trunk flexed; pendulum → horizontal abduction + scapular retraction.<br>5) Sitting: ipsilateral cervical side-bending.<br><br>C) Neurodynamics (accessory spinal nerve):<br>- Manual neurodynamic mobilization (by PT): side-lying on unaffected side; slight cervical flexion + scapular retraction; patient performs gentle cervical flexion↔neutral while therapist applies slight shoulder distraction + scapular depression (~10 minutes per session).<br>- Active neurodynamic exercises (10 reps each):<br>1) Standing: gentle cervical retraction (chin tuck) → neutral.<br>2) Standing: contralateral cervical rotation + flexion with ipsilateral shoulder abduction/extension and wrist flexion (arm straight).<br>3) Standing: fingers interlaced; maximal shoulder flexion, then neck flexion; hold ~3 s; release to pendulum with ~45° trunk flexion.<br>4) Standing: shoulder abduction ~50–70°; elbow flexion while bringing cervical spine to ipsilateral lateral flexion.
**5) Who provided**	Specialized physiotherapists delivered all sessions/interventions.	Specialized physiotherapists delivered all sessions/interventions.
**6) How (mode of delivery)**	Face-to-face, individually supervised sessions with therapist-instructed exercises.	Face-to-face, individually supervised sessions with manual therapy components

		and therapist-instructed exercises (mirror feedback).
**7) Where (setting)**	Clinical setting	Clinical setting
**8) When and how much (schedule, dose, duration)**	2 sessions/week for 8 weeks; 10 exercises × 20 repetitions each per session.	Active exercises: 10–12 reps each; manual neurodynamics: ~10 minutes per session.<br>2 sessions/week for 8 weeks
**9) Tailoring (what/how)**	Mirror used to minimize compensations during active exercises.	Mirror used to minimize compensations during active exercises.
**10) Modifications (during the study)**	None	None
**11) How well — planned (fidelity strategies)**	Therapist supervision	Therapist supervision
**12) How well — actual (adherence/fidelity achieved)**	Therapist supervision	Therapist supervision
